# Bony Metastasis of Gastric Adenocarcinoma

**DOI:** 10.4103/1319-3767.49009

**Published:** 2009-04

**Authors:** Bhavesh Devkaran, R. Jhobta, D. K. Verma

**Affiliations:** Department of Surgery, Indira Gandhi Medical College, Shimla - 171 001, Himachal Pradesh, India. E-mail: devkaranbhavesh@yahoo.co.in

Sir,

A 50-year-old male presented with history of abdominal pain, anorexia, early satiety, significant weight loss, and features of gastric outlet obstruction for 3 months. There was a history of maelena and hemetemesis. There was a history of dull aching pain in the right thigh for the last 2 weeks. Examination revealed marked pallor and a huge intra abdominal lump in the epigastric region extending to right hypochondrium and umbilical region. There was a hard swelling of size 5 × 4 cm on the medial aspect of right mid thigh.

**Figure 1:**
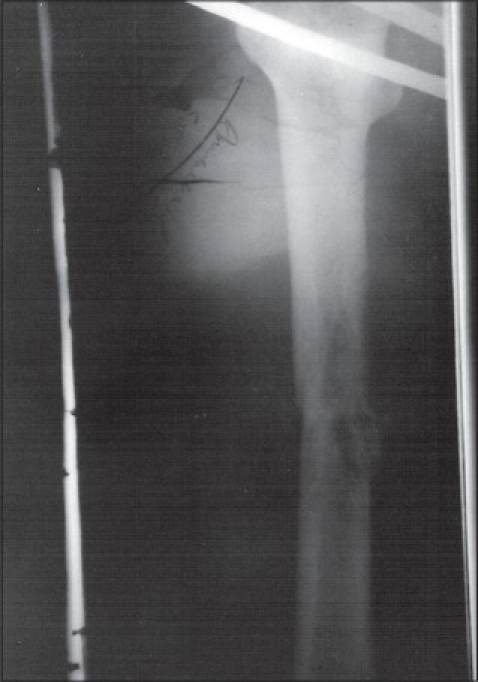
Pathological fracture femur

Upper gastrointestinal endoscopy revealed a large polypoidal mass in body of stomach extending to fundus at least 5 cm distal to gastroesophageal junction. Computed tomography abdomen showed a heterogenous density exophytic mass arising from medial wall of body of stomach. X-ray of right thigh showed an osteolytic lesion in the midshaft region of femur [Figure 1]. Biopsies obtained during endoscopy and fine needle aspiration cytology of soft tissue swelling of the right thigh revealed moderately differentiated adenocarcinoma.

Patient was explored and feeding jejunostomy done as growth was unresectable. On 10^th^ postoperative day, patient developed fracture of the shaft of the femur for which internal fixation was done. Patient was given single fraction radiotherapy along with chemotherapy (5-FU and leucoverin) and discharged.

Gastric carcinoma is a frequent tumor, especially in some parts of the world like Japan. Metastasis to the bone from gastric tumors is rare and has been estimated to appear in 13.4% of the autopsy cases of gastric carcinoma in a Japenese study.[1] It mainly affects patients with poorly differentiated tumors and widespread disease along with metastasis to other sites. However, there have been reports of bony metastasis from early gastric cancer. Metastasis to the bone can occasionally be the first manifestation of gastric tumor,[2] but have only rarely been described as a sole manifestation of tumor recurrence.[3]

Skeletal metastatic lesions arising from gastric cancer are uncommon and usually of osteolytic type. The thoracic and lumbar vertebrae are the most frequent sites[2] although there have been occasional reports of metastasis to the calcaneal bone,[3] pelvis, and even the skull base. Metastasis to femur is very rare.[2,3] Radioisotope bone imaging is generally accepted as being the initial procedure of choice in search for bone metastasis. Roentgenographic evaluation for bone metastasis has limited value because symptoms from bone metastasis frequently occur before any radiological abnormality becomes evident.

Prognosis of patients with osseous metastasis from gastric cancer seem to be dismal (median survival time 5 months) and 3.5 years has been the longest survival period reported in the literature. Radiotherapy has been advocated as the best therapeutic alternative for pain control with a response rate of 75%.[4] However, recent reports have employed chemotherapy with 5-FU with no adverse side effects. Nevertheless, prognosis remains poor and therapy is mainly aimed at relieving pain and discomfort.
